# THE ROLE OF GENDER IN THE RELATIONSHIP BETWEEN VIOLENCE EXPOSURE AND PSYCHOLOGICAL DISTRESS IN MIDDLE AGE

**DOI:** 10.1093/geroni/igae098.3440

**Published:** 2024-12-31

**Authors:** Khalil Iktilat, Michal Isacson, Roy Tzemah-Shahar, Maayan Agmon

**Affiliations:** Haifa University, Dabburiya, HaZafon, Israel; Massachusetts Institute of Technology, Cambridge, Massachusetts, United States; University of Haifa, Haifa, HaZafon, Israel; University of Haifa, University of Haifa, HaZafon, Israel

## Abstract

Middle age is a critical period in the lifecycle, with unique dynamics in men and women respectively. It represents a sensitive window for health prevention that can improve the aging process. To date, it remains unclear how environmental factors, like exposure to violence, influence psychological distress in this age group. In other age groups, women were observed to develop heighter distress than men following violence exposure. Here we aim to examine this gender-specific association between levels of exposure to violence and psychological distress in a population of middle-aged Muslims in Israel, who have been exposed to increasing violence in the past decade. A cohort of 363 middle-aged adults (223 women) from three Muslim villages in northern Israel was recruited. Psychological distress was assessed by the Kessler 6 Psychological Distress questionnaire, and violence exposure was assessed by the Screen for Adolescent Violence Exposure (SAVE) questionnaire. We controlled for age, sex, education level, and socioeconomic status using multivariate linear regression models with post-hoc models for each gender separately. Middle-aged males reported significantly greater exposure to violence whereas women reported significantly heightened psychological distress levels. A positive link between exposure to violence and psychological distress (β=0.145, p=0.017) was found using a multivariate model, and sex was a significant covariate (β=0.132, p=0.045). Post-hoc modelling revealed that this relationship was only significant among women (β=0.160 p=0.041). Our findings demonstrate that middle-aged women are at risk for violence-associated psychological distress. This research provides evidence for gender-based prevention and intervention programs.

